# Prognostic value of B cells in cutaneous melanoma

**DOI:** 10.1186/s13073-019-0647-5

**Published:** 2019-05-28

**Authors:** Sara R. Selitsky, Lisle E. Mose, Christof C. Smith, Shengjie Chai, Katherine A. Hoadley, Dirk P. Dittmer, Stergios J. Moschos, Joel S. Parker, Benjamin G. Vincent

**Affiliations:** 1 Lineberger Comprehensive Cancer Center, The University of North Carolina at Chapel Hill, Chapel Hill, NC 27599 USA; 2 Department of Microbiology and Immunology, The University of North Carolina at Chapel Hill, Chapel Hill, NC 27599 USA; 3 Department of Genetics, The University of North Carolina at Chapel Hill, Chapel Hill, NC 27599 USA; 4 Department of Medicine, The University of North Carolina at Chapel Hill, Chapel Hill, NC 27599 USA

**Keywords:** Immunology, Cancer, B cells, Machine learning, Melanoma

## Abstract

**Background:**

Measures of the adaptive immune response have prognostic and predictive associations in melanoma and other cancer types. Specifically, intratumoral T cell density and function have considerable prognostic and predictive value in skin cutaneous melanoma (SKCM). Less is known about the significance of tumor-infiltrating B cells in SKCM. Our goal was to understand the prognostic and predictive value of B cell phenotypic subsets in SKCM using RNA sequencing.

**Methods:**

We used our previously published algorithm, V’DJer, to assemble B cell receptor (BCR) repertoires and estimate diversity from short-read RNA sequencing (RNA-seq). We applied machine learning-based cellular phenotype classifiers to measure relative similarity of bulk tumor sample gene expression profiles and different B cell phenotypes. We assessed these aspects of B cell biology in 473 SKCM from the Cancer Genome Atlas Project (TCGA) as well as in RNA-seq data corresponding to tumor samples procured from patients who received CTLA-4 and PD-1 inhibitors for metastatic SKCM.

**Results:**

We found that the BCR repertoire was associated with different clinical factors, such as tumor tissue site and sex. However, increased clonality of the BCR repertoire was favorably prognostic in SKCM and was prognostic even after first conditioning on various clinical factors. Mutation burden was not correlated with any BCR measurement, and no specific mutation had an altered BCR repertoire. Lack of an assembled BCR in pre-treatment tumor tissues was associated with a lack of anti-tumor response to a CTLA-4 inhibitor in metastatic SKCM.

**Conclusions:**

These findings suggest an important prognostic and predictive role for B cell characteristics in SKCM. This has implications for melanoma immunobiology and potential development of immunogenomics features to predict survival and response to immunotherapy.

**Electronic supplementary material:**

The online version of this article (10.1186/s13073-019-0647-5) contains supplementary material, which is available to authorized users.

## Background

The adaptive immune response plays a dynamic role during cancer development, progression, and metastases via a process called immune editing [1]. Skin cutaneous melanoma (SKCM), in particular, may result in the outgrowth of tumor clones that either lack (“non-inflamed” or “cold”) or show a strong association with tumor-infiltrating lymphocytes (“inflamed” or “hot”) [2]. Multiple lines of evidence support the importance of melanoma-specific effector T cells in mediating anti-tumor immunity. The degree of effector T cell responses, which may be directed against either self-antigens overexpressed by melanoma cells, cancer testis antigens, or neo-antigens derived from expressed somatic mutations in melanoma [3–5], has been both a favorable prognostic factor in SKCM patients [6, 7] and a predictor of response to immunotherapies in metastatic SKCM [8–11].

There is a limited but growing body of evidence that B cells play an important role in melanoma. For example, patients with metastatic SKCM can develop antibodies against cancer testis antigens [12, 13], and the presence of these antibodies has been associated with clinical benefit from CTLA-4 inhibition [13]. Patient-derived B cells can kill melanoma cells in vitro by antibody-dependent cellular cytotoxicity (ADCC) [14]. Depletion of B cells was associated with impaired T cell tumor infiltration and cytotoxicity in the murine B16 melanoma model [15, 16]. Analysis of 329 SKCM specimens as part of The Cancer Genome Atlas (TCGA) project showed that patients who were classified as “immune-high” based on RNA sequencing (RNA-seq) contained a pleomorphic immune infiltrate that consisted of various immune cell subsets in addition to T cells, including B cells [17]. In patients with metastatic SKCM, tumor-infiltrating B cells had increased B cell receptor (BCR) class switching and affinity maturation [18], suggesting the presence of an active antigen-driven B cell response. B cell infiltration was correlated with T cell infiltration and with improved prognosis in SKCM [6, 7, 19, 20]. Other prior work has shown that tumor associated B cells may be associated with poor prognosis, confer resistance to targeted therapy via induced expression of IGF-1, and promote tumor progression via increased angiogenesis and support of lymph node metastasis [21–25]. Thus, precise molecular descriptions of B cell biology in melanoma with utility in clinical prognosis and in particular the role of tumor-infiltrating B cells are underdeveloped.

Analyses of adaptive immune responses have been accelerated by next-generation sequencing-based (NGS) approaches to profile T cell receptor (TCR) and BCR repertoires [9, 26]. We recently analyzed RNA-seq data from multiple cancers as part of the TCGA project and reported that B cell receptor abundance and diversity of the BCR variable region (V-region) are independent prognostic factors with respect to overall survival in SKCM [7]. This work demonstrated the utility of whole transcriptome profiling to characterize the immune cell repertoire, but could not resolve more specific aspects of B cell biology, such as the prognostic significance of individual B cell subsets and BCR repertoire characteristics estimated from complete VDJ transcripts. The latter is now possible using our recently published bioinformatics tool, V’DJer, which reconstructs BCR sequences from bulk tumor RNA-seq data, and thereby quantifies the type and extent of the repertoire of tumor-infiltrating B cells [27]. Furthermore, analysis of tumor datasets with limited or no systemic treatment information, such as the TCGA project, cannot address the question about the importance of B cell response as predictors of response to immunotherapies in metastatic SKCM.

To address these gaps in our knowledge, we analyzed RNA-seq data from the complete TCGA SKCM tumor dataset (*n* = 473 tumors) [17] as well as publicly available RNA-seq data from tumor tissues that were collected prior to treatment with immune checkpoint inhibitors [8, 28]. Integrating results from BCR repertoire profiling, TCR repertoire profiling, and machine learning-based classifications, we determined the prognostic and predictive significance of B cells and the BCR repertoire in metastatic SKCM. We found that clonally restricted BCR repertoire measured using species evenness was a favorable prognostic factor in SKCM. Species evenness is a calculated by dividing Shannon entropy by the total number of species (clones) and measures uniformity of the observed species.

## Methods

### Patients, clinical data, and mutation classification from the TCGA cohort in cutaneous melanoma

Patient demographics (age and sex), stage, and tumor tissue site were downloaded from GDAC Firehose from June of 2016. The survival data used was from the curated TCGA survival data from Lui et al. [29].

The mutations from each specimen were annotated by the UNCseq™ pipeline (version 2016.07; see below “next generation sequencing - the TCGA cohort in Cutaneous Melanoma”). Each specimen was subsequently classified according to the previously reported molecular classifications [17]: BRAF V600/601, RAS-mutant (NRAS, KRAS, and HRAS), NF1-mutant (with high-impact mutation only), and triple “wild-type”.

### Applying TCGA molecular subtype classification to full RNA-seq data set

A gene expression-based classification was previously described that classified 329 TCGA samples into three groups called “immune-high”, “keratin-high”, and “MITF-low” [17]. To apply this to the final full TCGA cohort of 473, we developed a gene expression classifier based on the previously published data. We calculated the silhouette width scores for each of the original 329 samples using the 1500 genes from the original analysis [30]. Samples with a positive silhouette value (*n* = 263) were considered most representative of each subtype and used to develop a gene expression predictor with Classification to the Nearest Centroids (CLaNC) method [31]. The final predictor consisted of 1260 genes with a 7.2% cross validation error rate and a 5% training error rate. This predictor was applied to the entire cohort to predict subtypes on the additional TCGA samples.

### Assembly and analysis of BCR and TCR sequences

BCR sequences were assembled using the same parameters as we have previously described [27]. Diversity measures (described below) were computed based on outputs from V’DJer using divBCR (an R function that calculates count and diversity metrics from the V’DJer output, https://github.com/sararselitsky/divBCR). TCR repertoire was analyzed from paired-end FASTQ files via MiXCR v1.8.1 RNA-seq mode [32]. Alignment was performed using both default and RNA-seq modes, targeting all TCR loci.

### Diversity measures

The total count measure is the sum of the expression of all BCR clones normalized by total RNA-seq read count. Top clone proportion and second top clone proportion measure the proportion of the most expressed and second most expressed clone in a population, respectively. Mean V-region identity estimates somatic hypermutation by determining the mean of the number of nucleotides that vary from the International ImMunoGeneTics Information System (IMGT) [33]. We also calculated diversity indices commonly used in the ecology literature [34, 35], Shannon entropy and 1-Gini-Simpson (referred to as Gini-Simpson), which measure species richness (number of species, or sequences), abundance, and evenness of the species in a population. We also measured species evenness, which is Shannon entropy normalized by species richness (Additional file 1: Figure S4).

**Fig. 1:**
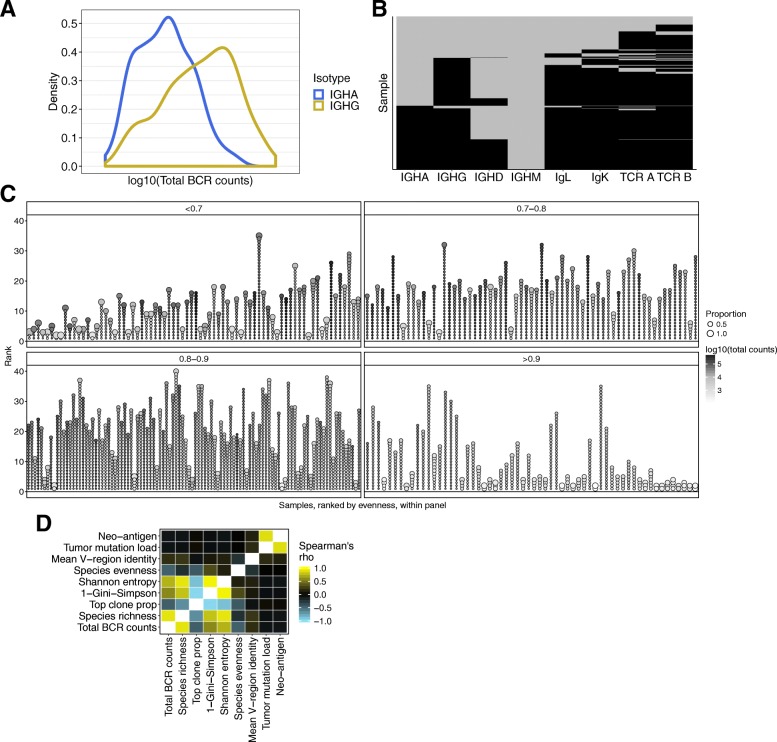
B cell receptor data. **a** Density plot of total BCR counts for IGHG (yellow) and IGHA (blue). **b** Assembled “chain” status for TCGA SKCM (*n* = 473). The samples are on the *y*-axis, each row is one sample, and chain types are on the *x*-axis. Black tiles indicate that a sample has at least one assembled sequence and gray is none. The samples are ordered by status for each chain type, from left to right on the *x*-axis. **c** Visual depiction of clonality for the entire TCGA melanoma cohort with a successfully assembled BCR (*n* = 337). *x*-axis displays each individual tumor sample. The *y*-axis is the rank of the proportion of each clone within a tumor. Each unique IGHG BCR sequence (clone) with a relative proportion > 0.01 is represented by a circle. The diameter of each circle represents the proportion of the sequence for each sample. The circles are ordered by proportion, rank, in descending order. Grayscale represents the total BCR counts. The samples are split into four sub-panels based on species evenness and ordered by low to high evenness within each sub-panel. **d** Pair-wise correlation heatmap. The color in each cell represents Spearman’s rank correlation coefficient of IGHG measurements correlated with IGHG measurements. Samples included in this analysis are TCGA SKCM samples with a value for each feature analyzed. See Additional file 2: Table S1. * *p* value < 0.05, ** *p* value < 0.01, *** *p* < 0.005, **** *p* value < 0.0005

See Additional file 1: FigureS3 for a demonstration of the diversity measures in toy examples. The BCR population structure for all TCGA melanoma samples is visualized in Fig. 1.

### Classification and subtype predictions

BAGS classifier (GSE56315 [36]) was built using Linear Distance Weighted Discrimination [37] (dwdLinear from the R package *Caret*) of genes with a standard deviation of log2 transformed RNA-seq > 0.2. IL10±-producing B cell classifier (GSE50895 [38]) was built same as BAGS, except it used all genes. For both of these, the classification subtype of each sample was called by the sub-classification with the highest probability.

### Statistical analysis

All statistical analyses and plots were generated using R version 3.4.1 (2017-06-30). Kaplan-Meier plot and Cox proportional hazards regression model were implemented using the R package *survival*. R packages used for analyses were *stats*, *plyr*, *reshape2*, and *caret.* R packages used for generating plots were *ggplot2* and *heatmap.plus.*

## Results

### B cell receptor repertoire in SKCM RNA-seq data

Immunoglobulin heavy chain genes are expressed in different isotypes (γ, δ, α, and μ), each indicative of a particular state of B cell differentiation and activation. Using V’DJer [27], a bioinformatics software tool that assembles BCRs (both heavy and light chains) from RNA-seq, we found that immunoglobulin heavy chain γ (IGHG, individual γ subclasses could not be distinguished), which is associated with an activated B cell response, was the most abundant heavy-chain isotype in the 473 SKCM samples (median read count of samples with an assembled sequence = 8301, Fig. 1a, b). This was followed by immunoglobulin heavy chain α (IGHA, median read count = 898.7), which is associated with a mucosal B cell response. In contrast, the immunoglobulin heavy chain μ (IGHM, median read count = 613.5) and immunoglobulin heavy chain δ (IGHD, median read count = 256.0) isotypes, which are associated with naïve B cells, were the least abundant antibody subtypes (Fig. 1b). The Igκ and Igλ light chains are coupled with any of the heavy chains, but since IGHG was the most abundant, they are most likely associated with IGHG. We are not able to couple the heavy and light chains in silico from bulk RNA-seq experiments*.*

As a quality control check, we correlated BCR repertoire estimates with total RNA-seq coverage and found no correlation (Additional file 1: Figure S1, Spearman’s rank rho (*ρ*) range 0.03–0.17), meaning the B cell signal was not a result of variation in read depth. V’DJer successfully assembled sequences of IGHA for 197 (42%), IGHG for 337 (71%), and IGHM for 117 (25%) out of 473 samples. Ninety-five percent of samples with an assembled IGHA also had an assembled IGHG, and 99% of samples with an assembled IGHD had an assembled IGHG (Fig. 1b). V’DJer also assembled sequences of the light Igκ chains for 348 (74%) and Igλ chains for 331 (70%) samples.

Due to the stringent read depth requirements (> 25× depth for 90% sensitivity) or lack of B cell presence, V’DJer failed to reliably assemble BCR sequences from any chain in 102/473 (21%) of samples. Samples where no IGHG was assembled demonstrated significantly lower read counts aligned to the IGHG constant region (*p* = < 2.2 × 10^− 16^ using the Mann-Whitney *U* test, Additional file 1: Figure S2), suggesting that lack of assembled BCR is due to low expression of the gene. We assessed assembled sequences corresponding to IGHA, IGHG, Igκ, and Igλ. Assembled IGHM and IGHD sequences occurred in too few samples for further consideration (*n* = 117 and *n* = 5 respectively, Fig. 1b).

We assessed several measurements of the BCR repertoire: total BCR counts (sum of all the counts from the assembled BCRs), top clone proportion, mean V-region identity (surrogate for somatic hypermutation, SHM), and three diversity measures: Shannon entropy, Gini-Simpson, and species evenness (see “Methods,” toy example shown in Additional file 1: Figure S3, Additional file 2: Table S1). Of the TCGA SKCM samples with assembled BCR sequences, there was considerable variation in the BCR populations identified; some samples showed evidence of clonal restriction, while others had high BCR diversity. Figure 1c depicts the BCR repertoire of all TCGA SKCM samples with an assembled BCR.

As expected [39], for IGHG BCR sequences, two diversity indices, Shannon entropy and Gini-Simpson, which are determined by the total number of clonotypes and their relative abundance, were strongly and significantly correlated with total BCR abundance (*ρ* = 0.80 and 0.69, respectively, *p* < 2.2 × 10^− 16^ by Spearman’s rank correlation, Fig. 1d). Species evenness, as an individual measurement, was significantly anti-correlated with total BCR abundance (*ρ* = − 0.47, *p* < 2.2 × 10^− 16^). Tumor somatic mutation burden and predicted neo-antigen burden did not correlate with any of the BCR repertoire measurements in these pre-treatment samples, suggesting that the B cell response was also directed against antigens not expressed within melanoma tumors (e.g., cancer testis and melanocyte differentiation antigens, or infectious agents) as well as possible melanoma-specific antigens.

### BCR associations with various clinicopathologic and molecular factors

We tested the clinical variables tumor tissue site, sex, age (as a binary, > 65 and < 65), and stage at diagnosis, for their association with the various BCR measurements using univariate linear regression and found that no BCR measurement was significantly associated with stage or age. In contrast, sex was significantly associated with multiple diversity measures. Females had significantly higher BCR diversity measured by both Gini-Simpson (*p* = 0.01 by the Mann-Whitney *U* test) and Shannon entropy (*p* = 0.02) than males, and correspondingly a lower top clone proportion (*p* = 0.01), but no significant difference in BCR abundance or evenness (*p* = 0.11, *p* = 0.74, respectively Additional file 1: Figure S4), suggesting that female melanoma patients may have a more diverse B cell response.

Tumor tissues analyzed in the TCGA SKCM dataset were acquired from primary tumors as well as tumors arising at different metastatic sites. We assessed if the B cell diversity metrics were altered based on site and found that diversity was lower in the primary tumor and regional subcutaneous metastatic samples (Shannon entropy *p* = 0.002, Gini-Simpson *p* = 0.02 by ANOVA, Additional file 1: Figure S5), and mean V-region identity was lower in the primary tumor (*P* = 4 × 10^− 9^). The higher SHM and lower diversity in the primary and regional metastatic samples could be due to the presence of more immature B cells in the distant metastatic sites, including lymph node metastases that would be expected to contain tissue resident B cells. However, total BCR counts and evenness were not significantly different (*p* = 0.06 and *p* = 0.4 respectively).

First, we tested the association of each IGHG BCR measurement with overall survival (OS) and found that species evenness, total BCR counts, Shannon entropy, and species richness were significantly prognostic (Table 1, *p* = 0.002, 0.01, 0.05, and 0.05, hazard ratio [HR] = 1.38, 1, 0.72, 0.85, respectively, Cox proportional hazards regression model). We next sought to determine whether BCR repertoire measurements were associated with OS after first accounting for the clinical variables tumor tissue site, sex, age at diagnosis, and stage. Species evenness and total BCR counts provided prognostic information in addition to the clinical variables (*p* = 0.001 and *p* = 0.04, respectively, log likelihood ratio test, HR = 1.45 and 0.72, respectively, Cox proportional hazards regression model). Overall, we found that more clonal expansion and higher B cell infiltrate was significantly associated with OS even after accounting for clinical variables.

**Table 1:** Univariate model: association of overall survival and each IGHG BCR measurement using Cox proportional hazard regression model. Multivariate model: association of overall survival and each IGHG BCR measurement after conditioning on clinical variables (tumor tissue site, sex, age at pathological diagnosis, and patient stage) using the log likelihood ratio test to determine *p* values and Cox proportional hazard regression model to determine the hazard ratio

	Univariable		Multivariable	
	Hazard ratio	*p* value	Hazard ratio	*p* value
Species evenness	1.38	0.0017	1.45	0.0014
Total BCR counts	0.72	0.0102	0.72	0.0104
Shannon entropy	0.85	0.0464	0.88	0.1727
Species richness	0.82	0.0512	0.83	0.0864
1-Gini-Simpson	0.87	0.0856	0.92	0.3852
Top clone prop	1.13	0.1407	1.07	0.4970
Mean V-region identity	0.94	0.4604	1.01	0.9256
Total mutation load	0.87	0.5624	0.75	0.3381
Neo-antigen	1.01	0.8997	1.00	0.9736

### Comparison of melanoma subtypes and machine learning-derived B cell phenotypes

Given that lower IGHG species evenness was a favorable prognostic factor in SKCM, we hypothesized that the presence of distinct tumor-infiltrating B cell subsets may be associated with BCR repertoires and outcomes. We classified TCGA SKCM samples based on their B cell subsets, by constructing two classifiers from publicly available datasets: B cell-associated gene expression signatures (BAGS, GSE56315) for five functionally different B cell subtypes (naïve, centrocytes, centroblasts, memory, and plasmablasts) [36], and a second classifier of B regulatory cells that exert an immunoregulatory role or not (GSE50895) [38]. It should be emphasized that this classification may not be measuring B regulatory cells, but more indicative of an immunosuppressive tumor microenvironment. Both classifiers were applied to score each SKCM sample with a relative measure of similarity to the known classes. These scores do not necessarily reflect the B cell population cellular phenotype distribution, but do measure the relative similarity between bulk tumor sample gene expression profiles and each of the B cell classes. These scores were then taken as relative measures of B cell subsets but may not truly be a measure of any B cell populations. The score for the B regulatory cells and BAGS memory classification were significantly prognostic after accounting for age, sex, tissue site, and stage (*p* = 4 × 10^− 9^, 2 × 10^− 8^ respectively, log likelihood ratio test, Additional file 1: Figure S6). The B regulatory cell score was associated with shorter OS, while the memory B cell score was associated with longer OS.

We next investigated whether any of the subgroups derived from the proposed TCGA classifications [17] on the basis of the most abundant somatic mutations (i.e., mutations in BRAF codons V600 and K601, RAS codons G12, G13, and Q61, and NF1 stop codon mutations) or gene expression profiling (melanogenesis associated transcription factor low (MITF-low), immune-high, and keratin-high) was associated with various BCR measures or other B cell subsets. Samples classified as memory B cells (mature, expanded and antigen-selected), and non-regulatory B cells, were strongly enriched in the TCGA immune-high subtype (chi-squared *p* < 2.2 × 10^− 16^, Additional file 1: Figure S7). There was no enrichment for either the BAGS classification or the B regulatory cell classification for any of the mutation classes.

**Fig. 2:**
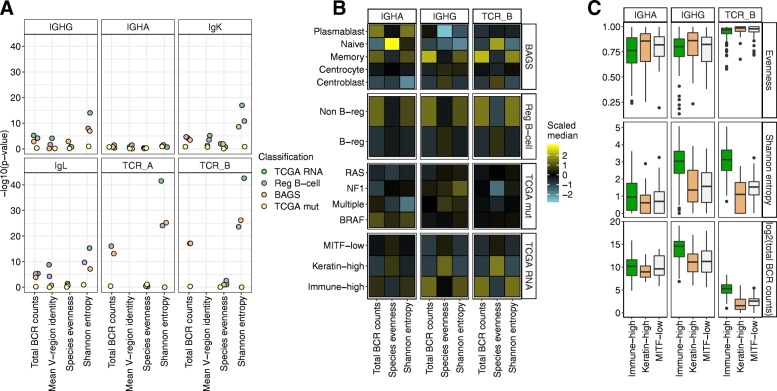
Variation of BCR and TCR measurements across different classifications. **a** *y*-axis, −log10(*p* value) determined using ANOVA separately, for four different classification types: TCGA RNA-seq molecular subtype (keratin-high, immune-high, MITF-low), reg B cell (IL-10± regulatory B cells), BAGS (B cell-associated gene signatures), and TCGA mut (status of BRAFV600/K601, RASG12/G13/Q61, and stop-codon NF1 somatic mutations). Each sub-panel is a different chain type. **b** Heatmap colored by scaled medians of each measurement across all subtypes. The plot is split into subplots by chain and classification. The medians were scaled across all sub-classifications, together. **c** Boxplots of selected BCR/TCR repertoire measurements separated by TCGA molecular subtypes split into sub-panels by immunoglobulin chain type. Boxes represent median ± interquartile range and whiskers ±1.5 × interquartile range. Outliers are represented by black dots. Samples included are TCGA SKCM samples with a value for each feature analyzed. See Additional file 2: Table S1

We performed an ANOVA to determine the variation across each of the TCGA tumor subtype’s BCR and TCR measurements (Fig. 2a). The TCGA molecular subtypes had the most variation across the different subtypes for the BCR diversity measurements (IGHG Shannon entropy, *q* = 1 × 10^− 14^, IGHG Gini-Simpson, *q* = 4 × 10^− 9^ by ANOVA after Benjamini-Hochberg multiple testing correction), indicating that tumor gene expression differences were associated with variation in B cell diversity (Fig. 2b, c). The BAGS and B regulatory cell classifications also significantly varied, but to a lesser degree (BAGS IGHG Shannon entropy, *q* = 1 × 10^− 7^; B regulatory cells IGHG Shannon entropy, *q* = 1 × 10^− 8^; Fig. 2a, b). Mutation status alone was not associated with any of the BCR diversity measurements.

### BCR and TCR features association in cutaneous melanoma

To gain insight into the relationship between tumor-infiltrating B and T cell populations, we assessed the T cell receptor (TCR) repertoire using MiXCR [32]. MiXCR was able to detect TCRs in > 80% of the SKCM samples. We estimated the TCR repertoire using the same metrics we applied to BCRs, except for V-region identity, since TCRs do not undergo somatic hypermutation. The abundance and diversity measures of the BCR repertoires were significantly correlated with those same measures for TCR repertoires (*ρ* = 0.47–0.55, *p* < 4 × 10^− 16^, Additional file 1: Figure S8), suggesting concordant activation of both cell types. Similar to BCR, TCR diversity and abundance were significantly associated with OS after accounting for the clinical variables tissue site, sex, age, and stage (Shannon entropy, *p* = 8 × 10^− 5^ and total TCR counts, *p* = 0.01, Cox proportional hazard’s model).

**Fig. 3:**
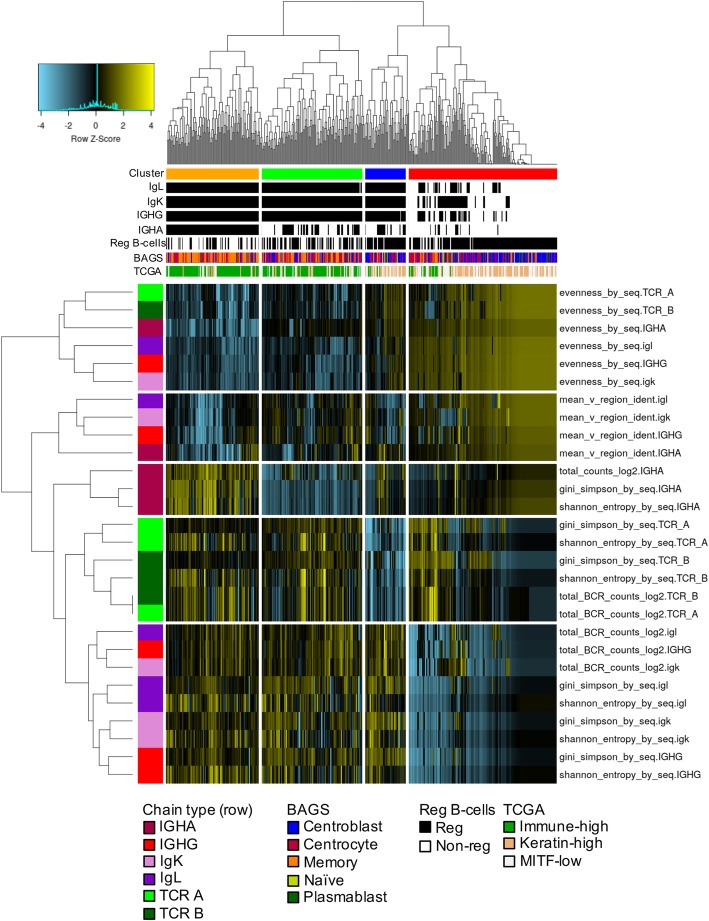
Unsupervised clustering of the TCGA SKCM samples according to BCR/TCR measurements. All TCGA melanoma samples (*n* = 473); samples with missing values were imputed for visualization purposes. For all BCR measurements missing values were replaced with a zero. Exceptions included evenness, which was replaced by one, and mean V-region identity, which was replaced by the median. Measurements were scaled and median-centered. Each row shows a BCR or TCR measurement, chain type represented by a different color in the row color bar. The columns correspond to samples. The column color bars represents the cluster assignment, the subtypes (TCGA RNA-seq, BAGS, B regulatory cell), and presence or absence of assembled chains

To assess the relationship between the B cell phenotype classifications and BCR and TCR repertoire measurements, we performed unsupervised hierarchical clustering (Fig. 3). Species evenness clustered together for all chain types, indicating an association between BCR and TCR restriction. Four clusters were identified. The orange and green clusters had higher BCR and TCR abundance, higher diversity, and lower species evenness and were enriched in the immune-high TCGA gene expression subtype, non-regulatory B cell subtype, and memory B cell subtype. These clusters had evidence of B and T cell clonal expansion and maturation, and they were split by expression of IGHA. The blue cluster, which had higher expression of IGHA and IGHG and lower expression of TCR-A and TCR-B, was enriched in IL10+ B regulatory cell and depleted of immune-high samples. The red cluster, which had low expression of both B and T cell expression, had an enrichment of centroblast and B regulatory cell-classified samples.

### B cell features are predictors of non-response to an immune checkpoint inhibitor in metastatic melanoma

**Fig. 4:**
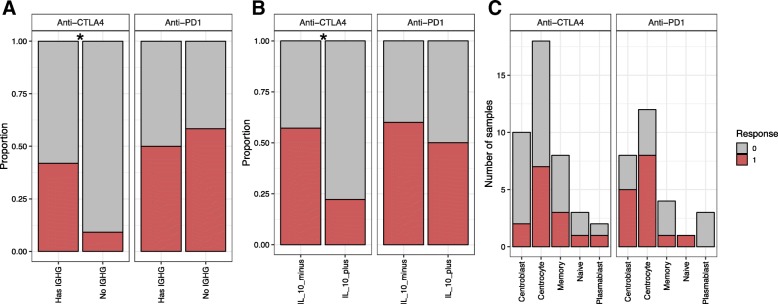
B cell associations with response to immunotherapy. The color represents the response status: gray represents non-responder and red represents responder. **a** Stacked bar plot showing the proportion of samples with an assembled IGHG (“has IGHG”) or no assembled IGHG. **b** Stacked bar plot showing the proportion of samples that were classified as IL10 secreting regulatory B cells (IL10_plus) or non-regulatory B cells (IL10_minus). **c** Stacked bar plot showing the number of samples that were classified into each of the different B cell subtypes from the BAGS classifier. * *p* value < 0.05

Density of tumor-infiltrating effector T cells has been previously associated with response to CTLA-4 and PD-1 inhibitors in SKCM [8, 28]; however, less is known about associations between B cells and immunotherapy response. We tested for differences in BCR repertoire diversity indices by response to treatment and found that none were significantly different in the responders compared to the non-responders (Additional file 1: Figure S9). However, the absence of an assembled IGHG was enriched in the non-responders (*p* = 0.04, Fisher’s exact one-sided test, Fig. 4a) for anti-CTLA4, but not anti-PD1. There were not enough samples without TCRs to test if the absence was associated with non-response. We tested if any TCR repertoire diversity measurement was associated with treatment response to either drug but found no significant association.

Next, we classified the anti-PD1 and anti-CTLA4-treated samples using the BAGS and B regulatory cell classifications. We found that non-responders to anti-CTLA4 therapy were enriched in the IL10+ classification (*p* = 0.03, Fisher’s exact one-sided test, Fig. 4b); however, there was no difference in response to anti-PD1. The IL10+ classification may be associated with an overall immunosuppressive microenvironment and not a true measure of the B cell subsets. There was no enrichment in either study for the BAGS classifications (Fig. 4c). Overall, we found that the absence of B cells and a tumor which more often classifies as an IL10 regulatory B cell were associated with a lack of response to anti-CTLA4 therapy.

## Discussion

Over the last 40 years, various reports have focused on the significance of T cells, in particular of the effector CD8 subtype, as both favorable prognostic factors and predictors of response to immunotherapies [40]. However, the density of tumor-infiltrating T cells alone is neither a prognostic factor for all cancers nor a sole predictor of response. We recently reported that B cell gene expression and BCR diversity were independently prognostic in TCGA SKCM and renal cancer by performing RNA-seq analysis using methods that assessed only the germline-only BCR region [7]. Detailed evaluations of full-length BCR sequences from short-read RNA-seq data have been limited due to inherent challenges in assembling immunoglobulin chains. In this report, we extend our observations on the specific B cell response as both a prognostic factor and possible predictor of response to CTLA-4 and PD-1 inhibitors in SKCM by integrating assembled BCRs determined from RNA-seq using V’DJer and machine learning-based classifications.

Our key observations are as follows: first, a highly abundant, clonally restricted BCR repertoire was a favorable prognostic factor in SKCM. Second, high-diversity, highly abundant BCR repertoire is not restricted to SKCM that bear high somatic mutation burden, suggesting that B cells can infiltrate melanoma in response to other antigens, such as melanoma differentiation and cancer testis antigens [17], viral antigens, and/or potentially other factors such as B cell chemotactic cytokines. This idea is further supported by the previous finding that women with SKCM have significantly lower somatic mutation burden than men [41] and our finding in this study that SKCM in women, who are known to have relatively decreased global central immune (i.e., thymic) tolerance and therefore more prone to autoimmune diseases [42], had a more diverse B cell infiltrate. Third, a substantial non-regulatory B cell presence is likely beneficial for response to anti-CTLA4 inhibitors.

Our study suggests a key role for tumor-infiltrating B cells in modulating the anti-tumor immune response. T cells can provide help to B cells, and B cells can secrete cytokines that support T cell proliferation and functional polarization. Since B cells also express immune checkpoint molecules such as CTLA-4, PD-1, and PD-L1 [43–46] that may negatively regulate BCR signaling [47], and since treatment with CTLA-4 or PD-1 inhibitors has been associated with increase in certain autoimmune antibodies [48, 49], it is possible that immune checkpoint inhibitors may enhance activation of B cells and overall contribute to either anti-tumor response (memory B cells) and/or development of autoimmunity [50]. In support of the former, our analysis of publicly available RNA-seq data corresponding to pre-treatment tumor tissues collected from patients who received CTLA-4 inhibitor suggests that lack of a B cell response is a predictor of poor response to immune checkpoint inhibitors.

Our results have other important clinical implications. We hypothesize that the greater clinical benefit seen in SKCM may be at least partially attributed to an important role of B cells in this disease. In fact, targeted therapies that can potentially inhibit B cell function if given at high doses, such as ibrutinib, which are currently in clinical development across various malignancies (NCT03021460, NCT02581930), may have undesirable clinical effects based on B cell inhibition. Analysis of peripheral blood for melanoma antibodies could complement other non-invasive studies to determine melanoma prognosis [14]. That said, there is also published data that B cell features can be associated with poor prognosis and tumor progression. Further work will be required to understand this heterogeneity and determinants of the complex roles of B cells in melanoma.

One limitation of our study is that tumor-infiltrating B cell subsets scored using BAGS classifiers were not validated using traditional tumor imaging techniques, such as immunohistochemistry or immunofluorescence. We were also not able to discriminate the proportion of B cells in each tumor sample that deeply infiltrated the tumor versus those that were tumor adjacent or physiologically present due to involvement of blood and/or lymphatic structures in the sample. Further studies will be needed to assess the localization of B cell populations within melanoma tumors and associate this with B cell functional phenotypes and B cell receptor repertoire profiles. While lack of this contextual validation is an inherent weakness across all TCGA projects where bulk gene expression profiling was used to infer immune features, it is important to emphasize that in the original report of the TCGA SKCM project the Pathology Analysis Working Group performed a semi-quantitative analysis on the density of tumor-infiltrating lymphocytes on hematoxylin and eosin-stained tissue sections, termed immune score, a method that inherently cannot discriminate between various lymphocytic cell subsets. Given that the OS is longer in patients with clonally restricted BCR repertoires, future tumor tissue-based translational studies in SKCM should include contextual assessment of BCR repertoire features and prognostically significant B cell subsets (memory, regulatory, IL-10 producing).

## Conclusions

In summary, we have shown that BCR repertoire measurements and B cell phenotypic population characteristics from RNA-seq data differ by SKCM gene expression subtype and associate with OS. The BCR repertoire was associated with different clinical factors, such as tumor tissue site and sex and increased clonality of the BCR repertoire was favorably prognostic in SKCM. The BCR repertoire was prognostic even after first conditioning on various clinical factors. Memory and IL10+-secreting B cell classifications were associated with prognosis (positive and negatively, respectively); however, these classifications were based purely on gene expression and need to be validated with orthogonal methods. Lack of an assembled BCR in pre-treatment tumor tissues was associated with a lack of anti-tumor response to a CTLA-4 inhibitor in metastatic SKCM. These findings suggest an important prognostic and predictive role for B cell characteristics in SKCM. These data have implications for understanding melanoma immunobiology as well as potential development of immunogenomics features to predict survival and response to immunotherapy.

## Additional files

Additional file 1: Supplementary figures. (DOCX 3697 kb)

Additional file 2: **Table S1.** All count/nucleotide/diversity measurements for each sample. Table S2 Classification information for each sample. (XLSX 560 kb)

## Data Availability

We used publicly available RNA-seq from TCGA. BAGS classifier was from gene expression data that can be accessed in GEO: GSE56315 [36]. IL10±-producing B cell classifier gene expression data can be accessed in GEO: GSE50895 [38]. Anti-CTLA4 sequencing data can be accessed in dbGap: phs000452.v2.p1 [8]. Anti-PD1 sequencing data can be accessed in GEO: GSE78220 [28].
