# Supplementary material for: Prognostic value of B cells in cutaneous melanoma
Source: Genome Med. 2019 May 28;11:36. doi: 10.1186/s13073-019-0647-5 (PMC6540526; doi:10.1186/s13073-019-0647-5)
Supplement: Supplementary file 1 — Supplementary figures. (DOCX 3697 kb) [file 13073_2019_647_MOESM1_ESM.docx]

**Fig S1.** Log10(total B/TCR counts) from V’DJer and MiXCR outputs plotted vs. log10(total RNA-seq) mapped reads of TCGA SKCM for IGHA (n=197), IGHG (n=337), IGHM (n=117), IgK (n=348), IgL (n=331), TCR alpha (n= 387), and TCR beta (n=412). Determined correlation coefficient (rho) using Spearman’s rank.

**Fig S2.** Density plots of constant regions for samples with and without an assembled B cell receptor (BCR), for IGHG, IGHA, and IGHM separately for all TCGA SKCM samples (n=473).

Tumor 1

Tumor 2

Tumor 3

|  | **Tumor 1** | **Tumor 2** | **Tumor 3** |
| --- | --- | --- | --- |
| Shannon entropy | 0.69 | 1.38 | 0.38 |
| Evenness | 1 | 1 | 0.54 |
| Gini-Simpson | 0.5 | 0.75 | 0.21 |
| Top clone proportion | 0.5 | 0.25 | 0.875 |

**Fig S3.** Toy example of B cell receptor measurements. Each circle represents a B cell and the color and thickness of the y on the circle (B cell receptor) represents a different sequence.

**Fig S4.** Boxplots of BCR repertoire features separated by sex. Boxes represent median ± interquartile range and whiskers ± 1.5 * interquartile range. Outliers are represented by black dots. Samples included in this analysis are TCGA SKCM samples with a value for each feature analyzed. See supplemental table 1. * indicates p-value <0.05.

**Fig S5.** Boxplots of BCR repertoire features separated by tissue site. Boxes represent median ± interquartile range and whiskers ± 1.5 * interquartile range. Outliers are represented by black dots. Samples included in this analysis are TCGA SKCM samples with a value for each feature analyzed. See supplemental table 1. * indicates p-value <0.05, ** p-value <0.01, *** indicates p<0.005, **** indicates p-value <0.0005.

|  | Univariable | | Multivariable | |
| --- | --- | --- | --- | --- |
|  | Hazard Ratio | P-value | Hazard Ratio | P-value |
| IL10+ | 1.50 | 1.12E-07 | 1.61 | 3.91E-09 |
| BAGS Memory | 0.67 | 1.38E-07 | 0.62 | 7.78E-09 |
| TCGA Immune | 0.68 | 1.43E-07 | 0.63 | 2.00E-08 |
| TCGA Keratin | 1.47 | 4.09E-09 | 1.47 | 2.65E-06 |
| BAGS Centroblast | 1.20 | 0.0032 | 1.26 | 0.0027 |
| BAGS Naive | 1.12 | 0.0629 | 1.26 | 0.0049 |
| TCGA MITF.low | 1.07 | 0.2902 | 1.16 | 0.0370 |
| BAGS Centrocyte | 0.89 | 0.0999 | 0.84 | 0.0790 |
| Mut, TripleWild | 1.19 | 0.0036 | 1.12 | 0.1078 |
| Mut, Multiple | 1.00 | 0.9818 | 0.90 | 0.1868 |
| Mut, RAS | 1.04 | 0.5671 | 1.09 | 0.2461 |
| BAGS Plasmablast | 0.94 | 0.3596 | 0.94 | 0.4333 |
| Mut, NF1 | 1.05 | 0.4316 | 0.96 | 0.5363 |
| Mut, BRAF | 0.90 | 0.1309 | 0.98 | 0.8343 |

**Fig S6.** Univariate model) association of overall survival and each subtype (all subtypes except mutation status are continuous) using Cox proportional hazard regression model. Multivariate model) association of overall survival and each subtype (all subtypes except mutation status are continuous) after conditioning on clinical variables (tumor tissue site, sex, age at pathological diagnosis and patient stage) using the log likelihood ratio test to determine p-values and Cox proportional hazard regression model to determine the hazard ratio.

**
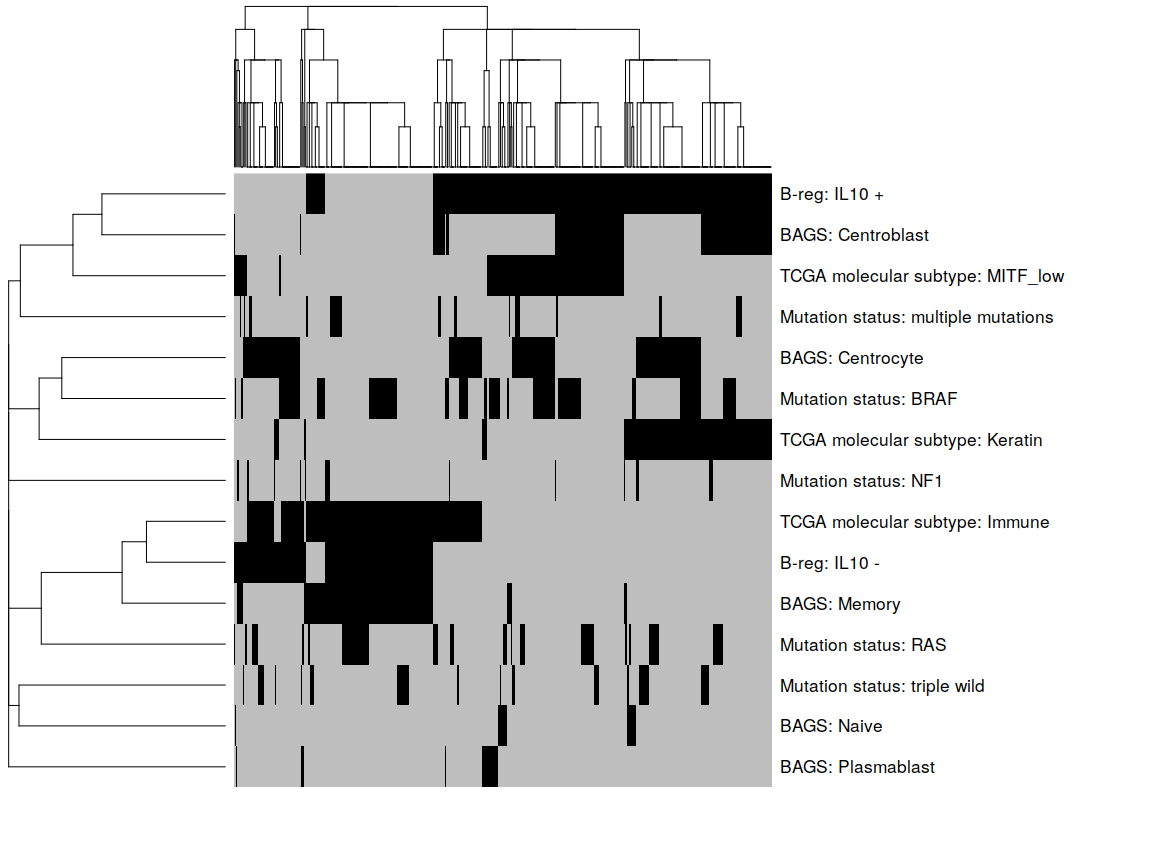
**

**Fig S7.** Heat-map of each sample’s binary classification status (gray: no, black: yes) clustered by hierarchical clustering of binary distance. See supplemental table 2.

**Fig S8.** Pair-wise correlation heat-map of BCR (IGHG) and TCR (TCR-B) repertoire features. The color in each cell in the heat-map represents the Spearman’s rank correlation coefficient. Samples included in this analysis are TCGA SKCM samples with a value for each feature analyzed. See supplemental table 1.

**Fig S9.** Boxplots of IGHG BCR measurement features separated by response status. Subplots representing the study and BCR repertoire measurement. Boxes represent median ± interquartile range and whiskers ± 1.5 * interquartile range. Outliers are represented by black dots. Samples included in this analysis are TCGA SKCM samples with a value for each feature analyzed. See supplemental table 1.
